# Low miR-936-mediated upregulation of Pim-3 drives sorafenib resistance in liver cancer through ferroptosis inhibition by activating the ANKRD18A/Src/NRF2 pathway

**DOI:** 10.3389/fonc.2024.1483660

**Published:** 2024-10-24

**Authors:** Xiao Li, Mengna Cui, Long Xu, Qie Guo

**Affiliations:** Department of Clinical Pharmacy, The Affiliated Hospital of Qingdao University, Qingdao, Shandong, China

**Keywords:** liver cancer, sorafenib, Pim-3, miR-936, ferroptosis

## Abstract

**Objective:**

Sorafenib, a multikinase inhibitor, is currently the standard treatment for advanced liver cancer. However, its application has become limited by the development of drug resistance. We intended to explore the mechanisms underlying the development of sorafenib resistance, therefore identifying an effective strategy to overcome sorafenib resistance remain challenges.

**Methods:**

Here, the follow-up of liver cancer patients undergoing sorafenib therapy, as well as animal tumor challenge and treatment were performed. The sorafenib-resistant liver cancer cell lines Huh7/SOR and HepG2/SOR were also established. miRNA and mRNA microarray analyses, TargetScan prediction, dual luciferase reporter assay, RNA pull-down assay, co-mmunoprecipitation (Co-IP) and pull-down assays, a transcription factor-specific NRF2 assay, an iron detection assay, a lipid peroxidation quantification assay, a ROS measurement assay, and GSH/GSSG and GSH-px standard quantitative assays were used.

**Results:**

We showed that upregulation of the provirus-integrating site for Moloney murine leukemia virus 3 (Pim-3) predicted poor response and unsatisfactory prognosis in sorafenib-treated liver cancer patients. Similarly, Pim-3 expression was positively associated with sorafenib resistance in liver cancer cells. Furthermore, microRNA-936 (miR-936) targeted the 3’-noncoding region (3'-UTR) of Pim-3 but exhibited lower expression in sorafenib-resistant liver cancer cells than in their parental cells. The high expression of Pim-3 mediated by miR-936 insufficiency activated the ANKRD18A/Src/NRF2 pathway which rearranged the expression of the indicated markers involved in iron distribution and lipid peroxidation homeostasis. MiR-936 overexpression and GV102-Pim-3-shRNA significantly attenuated the activity of the ANKRD18A/Src/NRF2 pathway to decrease the expression of Ankyrin repeat domain-containing protein 18A (ANKRD18A), Src, and Nuclear factor (erythroid-derived 2)-like 2 (NRF2), especially decreasing NRF2 nuclear retention and transcriptional activity. The transcriptional activity of NRF2 prompted cell ferroptosis because the transfection of miR-936 mimics, GV102-Pim-3-shRNA and GV102-NRF2-shRNA plasmid increased the expression of transferrin receptor 1 (TFR1) and divalent metal transporter 1 (DMT1) but decreased the expression of solute carrier family 7 member 11 (SLC7A11), glutathione peroxidase 4 (GPX4), quinone oxidoreductase 1 (NQO1), and heme oxygenase-1 (HO-1), thus facilitating the accumulation of intracellular Fe^2+^, lipid peroxides, and reactive oxygen species (ROS) but reducing the glutathione (GSH) level. Moreover, the elevated expression of Pim-3, resulting from the absence of miR-936 enhances sorafenib resistance in liver cancer by inhibiting cell ferroptosis.

**Conclusion:**

Pim-3 can be regarded as a target in the treatment of sorafenib-resistant liver cancer.

## Introduction

Liver cancer, more specifically hepatocellular carcinoma (HCC), ranks as the sixth most serious cancer and the fourth leading cause of cancer death worldwide (1). Surgery to remove the cancer and a margin of healthy tissue that surrounds it may be an option for patients with early-stage liver cancer who have normal liver function (2). Unfortunately, most liver cancer patients are diagnosed at an advanced stage, and therefore resection is not feasible (3). Systemic therapy based on multikinase inhibitors, chemotherapeutic drugs, and immune checkpoint inhibitors is now routinely prescribed to patients with advanced HCC (4). Rapid progress in the cornerstone treatment for advanced HCC has been made following the demonstration of a survival benefit with sorafenib, which has become the standard therapeutic drug for many patients with advanced liver cancer (5, 6). Other multikinase inhibitors, including lenvatinib, regorafenib and cabozantinib, have also shown substantial efficacy in advanced HCC (7). Immune checkpoint inhibitors such as atezolizumab, nivolumab, sintilimab and camrelizumab are also recommended as agents for treating HCC (8). However, sorafenib in particular has emerged as the most common systemic therapy for advanced HCC. Recent studies have highlighted the potential role played by ferroptosis in the sorafenib-induced therapeutic response in HCC. Indeed, promising evidence from some studies have indicated that sorafenib exerts its cytotoxic effects by triggering cell ferroptosis (9–13).

Ferroptosis, a newly recognized type of regulated cell death (RCD), occurs through the production of reactive oxygen species (ROS) from accumulated iron and lipid peroxidation (14). This type of cell death is still poorly understood ; however, evidence indicates that it is certainly distinct from apoptosis, necrosis and autophagy, and is characterized mainly by smaller-than-normal mitochondria with a condensed mitochondrial membrane and a lack of cristae (15). Specifically, ferroptosis activation is associated with iron overload favored by iron-responsive element binding protein 2 (IREB2), which increases transferrin receptor 1 (TFR1) and divalent metal transporter 1 (DMT1) expression but decreases ferritin heavy chain 1 (FTH1) expression (16, 17). Ferroptosis is also characterized by glutathione peroxidase 4 (GPX4) depletion caused by a reduction in the glutathione (GSH) level, which is guided by a reduction in solute carrier family 7 member 11 (SLC7A11) expression (18). Finally, a large quantity of ROS is produced via the *Fenton reaction* due to excessive iron. But lipid peroxides which are another source of ROS, cannot be eliminated due to GPX4 shortage, thus driving ferroptosis execution (19, 20). Nuclear factor (erythroid-derived 2)-like 2 (NRF2) is recently identified as a central moderator that maintains intracellular redox homeostasis (20, 21). NRF2 is also provided with the ability of scavenging lipid peroxides and ROS and pumping iron out of the cell to retard ferroptosis by regulating the GSH antioxidant system and the expression of iron metabolism genes (22–26). Activated NRF2 with enhanced transcriptional activity also prevented the accumulation of Fe^2+^ by activating the gene transcription of FTH1 and heightened the expression of heme oxygenase-1 (HO-1) and quinone oxidoreductase 1 (NQO1), which can scavenge lipid peroxides and ROS (27, 28).

Currently, many liver cancer patients tend to develop an obvious resistance to sorafenib, and thus the overall efficacy of the drug is unsatisfactory for these individuals (29). However, the implications of ferroptosis in the context of sorafenib resistance in liver cancer remain unclear.

The proviral integration site for the Moloney murine leukemia virus (PIM) kinases, including Pim-1, Pim-2, and Pim-3, have been implicated in cancer progression as oncogenic serine/threonine kinases (30). In particular, Pim-1 and Pim-2 are well understood to facilitate tumor survival, metastasis, and therapeutic resistance (31). However, few reports have described the protumorigenic roles of Pim-3 in HCC (32). We also previously provided compelling evidence that Pim-3 interrupts cell apoptosis by phosphorylating the proapoptotic BH3-only protein Bad, which may be a putative target for HCC treatment (33). However, much less is known about the role of Pim-3 in promoting sorafenib resistance in liver cancer.

Herein, we demonstrated that Pim-3 endowed liver cancer patients with insensitivity to sorafenib and predicts an undesirable prognosis in liver cancer patients receiving sorafenib treatment. Pim-3 expression was also particularly greater in sorafenib-resistant liver cancer cell lines Huh7/SOR and HepG2/SOR compared with the corresponding parental cells. Pim-3 knockdown significantly improved sorafenib sensitivity in Huh7/SOR and HepG2/SOR cells. However, exogenous Pim-3 expression wrecks the sensitivity of Huh7 and HepG2 cells to sorafenib. Furthermore, microRNA-936 (miR-936) inhibited Pim-3 expression by targeting its 3’-noncoding region (3’-UTR) but was rarely expressed in Huh7/SOR and HepG2/SOR cells. The aberrant expression of Pim-3 induced by a reduction in miR-936 expression, accelerated activation of the ANKRD18A/Src/NRF2 pathway, resulting in iron overload and lipid peroxidation, both of which are beneficial to ferroptosis, which was consequently lost in Huh7/SOR and HepG2/SOR cells. Transfection with miR-936 mimics and GV102-Pim-3-shRNA plasmid perturbed the activation of the ANKRD18A/Src/NRF2 pathway and reduced ANKRD18A, Src, and NRF2 expression, resulting in decreased NRF2 nuclear retention and transcriptional activity. Subsequently, miR-936 mimics and gene silencing of Pim-3 and NRF2 upregulated TFR1 and DMT1, but downregulated SLC7A11, GPX4, NQO1, and HO-1 expression; thereby increasing the levels of intracellular Fe^2+^, lipid peroxides, and ROS but decreasing the GSH level to promote cell ferroptosis. Most importantly, the aberrant expression of Pim-3 caused by miR-936 deficiency accelerated the development of sorafenib resistance by inhibiting cell ferroptosis in liver cancer.

These findings suggest that Pim-3 could serve as a putative target for reversing sorafenib resistance in liver cancer patients.

## Materials and methods

### Cell culture

The HCC lines including Hep3B (Catalog No.: SCSP-5045), Huh7 (Catalog No.: SCSP-526) and HepG2 (Catalog No.: SCSP-510), and human normal hepatocyte cell line THLE-2 (Catalog No.: SCSP-5068) were acquired from the Cell Bank of the Chinese Academy of Sciences (Shanghai, China). 293T cells (ATCC number: CRL-3216) and HEK-293 (ATCC number: CRL-1573) cells were purchased from the American Type Culture Collection (ATCC) (Rockville, MD, USA). These cells were maintained in RPMI 1640 medium (Gibco, Grand Island, NY, USA) supplemented with 10% heat-inactivated fetal bovine serum (FBS) (Gibco/BRL). THLE-2 cells were cultured in *Complete Growth Mediumin* provided by BEGM kit (Lonza/Clonetics, Catalog No.: CC-3170, Cell Bank of the Chinese Academy of Sciences). All of these cells were cultured at 37°C in a humidified atmosphere containing 5% CO2.

### Reagents, chemicals and antibodies

Sorafenib (CAS No.: 284461-73-0) and ferrostatin-1 (CAS No.: 347174-05-4) were purchased from Sigma-Aldrich. The miR-936 mimics and miR-936 inhibitor (anti-miR-936) were designed via the miRBase Sequence Database (http://microrna.sanger.ac.uk) and synthesized by GenePharma Co.,Ltd (Shanghai, China). Cel-miR-239b was used as the negative control and also achieved from GenePharmaCo.,Ltd (Shanghai, China). The following antibodies for Western blot assay were used: anti-human Pim-3 antibody (1:500 dilution, #ab198842, Abcam), anti-human GPX4 antibody (1:500 dilution, #ab125066, Abcam), anti-human NQO1 antibody (1:500 dilution, #ab80588, Abcam), anti-human TFR1 antibody (1:1000 dilution, #ab214039, Abcam), anti-human HO-1 antibody (1:500 dilution, #ab305290, Abcam), anti-human DMT1 antibody (1:500 dilution, #ab55812, Abcam), anti-beta actin antibody (1:1000 dilution, #BF0198, Affinity Biosciences), anti-human SRC antibody (1:500 dilution, #ab109381, Abcam), anti-human NRF2 antibody (1:500 dilution. #ab62352, Abcam), anti-human SLC7A11 antibody (1:500 dilution, #ab307601, Abcam), and anti-human ANKRD18A antibody (1:300 dilution, NDC-ASJ-XVKXAK-50, Nordic BioSite).

### Plasmid construction and lentiviral packaging

siRNA duplexes targeting Pim-3, SRC, and NFE2L2 (sense-loop-antisense) with overhanging ends identical to those created by restriction enzyme digestion (BamHI at the 5’ end and EcoRI at the 3’end) were designed via the BLOCK-iT RNAi Designer and then synthesized by Sangon Biotech Co., Ltd. (Shanghai, China). Alternatively, the complete coding sequences (CDSs) of Pim-3, ANKRD18A, and NFE2L2 were searched in the Nucleotide Sequence Database. DNA was extracted from HEK-293 cells and used as a template to amplify Pim-3, ANKRD18A, and NFE2L2. The pTZU6+1, hU6-MCS-CMV-GFP-SV40-Neomycin (GV102), and pcDNA3.1-CMV-MCS-3flag-EF1A-zsGreen-sv40-puromycin (GV657) vectors were purchased from GeneChem Co., Ltd. (Shanghai,China) and double digested with BamHI/HindIII and SacI/AgI, respectively. The polymerase chain reaction (PCR) fragments were recovered. GV102-Pim-3-shRNA, pTZU-SRC-shRNA, GV102-NRF2-shRNA, GV657-NRF2, GV657-Pim-3, and GV657-ANKRD18A vectors were constructed by ligation of the recovered fragments, transformation of ligation products, and identification by sequencing. The miR-936 mimics, miR-936 inhibitor, and the indicated vectors were transfected using Lipofectamine™ 3000 transfection reagent. The GV102-Pim-3-shRNA plasmid was constructed by cloning of the intended sequence and insertion into the pGCSIL-GFP vector, and the constructed vector was transfected into 293T cells. After 48 h, the cultured cells were centrifuged to collect harvested viruses containing the GV102-Pim-3-shRNA plasmid.

### Human samples

64 patients with primary liver cancer who underwent sorafenib therapy were followed up to evaluate their outcome, represented as overall survival (OS). The therapeutic effects of sorafenib on selected patients were evaluated according to the World Health Organization response criteria for solid tumors, and the grouping of patients was based on the evaluation results (the sensitive group consisted of patients with complete or partial remission, and the resistant group including the patients with progressive-stage tumors). The tissue samples collected from these patients were embedded in paraffin for immunohistochemical assays or preserved in liquid nitrogen. This study was approved by the Ethics Committee of the affiliated hospital of Qingdao University and written informed consent was obtained from each patient.

### Establishment of Huh7/SOR and HepG2/SOR cells

The sorafenib-resistant liver cancer cell lines Huh7/SOR and HepG2/SOR were established *in vitro* using the continuity induction method *in vitro*. Briefly, Huh7 and HepG2 cells in the logarithmic growth phase were seeded in culture medium containing sorafenib at an initial concentration of 0.1 µmol/L. Every two days thereafter, surviving cells were collected and cultured with fresh medium containing a higher concentration of sorafenib (increasing for 0.1 µmol/L each passage). The construction of Huh7/SOR and HepG2/SOR cells was completed over a 3-month period. The IC50 values of sorafenib in Huh7/SOR and HepG2/SOR cells as well as the corresponding parental cells were calculated via previously described methods (34). According to the calculation results, the IC50 values of sorafenib in Huh7/SOR and HepG2/SOR cells were 11.3 μM and 13.2 μM, respectively. In contrast, the IC50 values of sorafenib in the parental Huh7 and HepG2 cells were 4.0 μM and 3.6 μM, respectively.

### CCK-8 assay

Huh7/SOR and HepG2/SOR cells were pretransfected with the GV102-Pim-3-shRNA plasmid for 24 h using the Lipofectamine 3000 (Catalog No.:L3000001, Thermo Fisher Scientific), and then treated with sorafenib at increasing concentrations (0, 2.5, 5, 10, 15 and 20 μM) for another 48 h or with a constant concentration of sorafenib (10 μM) for 0-72 h. Alternatively, Huh7 and HepG2 cells were pretransfected with the GV657-Pim-3 plasmid for 24 h prior to treatment with sorafenib (0, 2, 4, 6, 8 and 10 µM) for another 48 h. For each group, 100 μl of drug-free medium containing 10% CCK-8 (#ab228554, Abcam) was added to each of three replicate wells. The cells were incubated for 2 h in an incubator containing 5% CO2 at 37°C, after which the optical density (OD) of the cells at 450 nm was measured. Cell viability was calculated as=[(As-Ab)/(Ac-Ab)]*100%, where As is the absorbance of experimental wells (containing cells, medium, CCK-8 solution and drug solution); Ac is the absorbance of control wells (containing cells, medium, and CCK-8 solution but no drug); and Ab is the absorbance of blank wells (containing medium and CCK-8 solution but no cells or drugs). The average percentage inhibition at each concentration was determined using previously described methods (34).

### Dual luciferase reporter assay

The 3’-UTR of the Pim-3 mRNA was PCR-amplified and inserted into the region directly downstream of the SV40 promoter-driven Renilla luciferase cassette in the psiCHECK-2 plasmid (GeneChem, Shanghai). The mutant (Δ) 3’-UTR of Pim-3 containing a point-mutated sequence in the miR-936 seed region was also constructed from the wild-type Pim-3 3’-UTR plasmid. Next, Huh7/SOR and HepG2/SOR cells were cotransfected with the miR-936 mimics and the psiCHECK-2 vector, and luciferase activity was determined using a Dual Luciferase Assay System (Promega).

### Chorion specific transcription factor NRF2 assay

The transcriptional activity of NRF2 was examined using the NRF2 Transcription Factor Assay Kit (Colorimetric) (#ab207223, Abcam), according to the manufacturer’s instructions.

### Lipid peroxidation assay

The lipid peroxide content, represented by the relative malondialdehyde (MDA) concentration, was determined using a Lipid Peroxidation Assay Kit (#ab118970, Abcam), according to the manufacturer’s instructions.

### ROS measurement

ROS levels were detected with the DCFH-DA Detection Kit (CAS No.:#CK0073, Signalway Antibody).The DCFH-DA probe (1:1000 dilution) was prepared in a serum-free medium. The indicated cells were collected and incubated with DCFH-DA probe at 37°C for 20 min. The fluorescence intensity of DCF was measured at excitation and emission wavelengths of 485 nm and 530 nm, respectively, using a fluorescence plate reader (Genios, TECAN).

### Iron assay

Iron levels were evaluated using an Iron Assay Kit (Colorimetric) (#ab83366, Abcam), according to the manufacturer’s instructions.

### GSH/GSSG and GSH-px standard quantitative assay

The intracellular levels of GSH, GSSG, and GSH-Px were assessed with the GSH/GSSG Assay Kit (commercial, #S0053) and the GSH-Px Assay Kit (commercial, #S0056), which were purchased from Beyotime Biotechnology, in accordance with the manufacturer's specifications.

### Animals, tumor challenge and treatment

Huh7 cells were inoculated into the left armpit of female nude mice (5-6 weeks old). After 3 weeks of tumor growth, tumor-bearing mice were grouped (four or five nude mice per group) to receive different treatments: (1) the control group, which was treated with PBS; (2) the sorafenib monotherapy group, which was administered sorafenib (30 mg/kg) by intraperitoneal injection every three days for a total of three weeks; and (3) the combination group, which was managed with alternative treatment of sorafenib and the indicated plasmids. Tumor-bearing mice were intratumorally injected with LV-Pim-3-shRNA vector via the tail vein. Sorafenib (30 mg/kg) was administered via intraperitoneal injection. This cycle was alternated every three days for three weeks. The mice were sacrificed, and the tumor volume and weight were measured. All experimental procedures were approved by the Animal Care and Use Committee of the affiliated hospital of Qingdao University.

### Real-time PCR assay

RNA from the indicated cells or tissues was extracted using TRIzol reagent (CAS No.: 15596026CN, Invitrogen). To extract miR-936, cel-miR-39 mimics were added as external standards. MiR-936 was isolated using a MiRcute miRNA Isolation Kit (CAS No.: DP501,Tiangen Biotech Co.,Ltd.). The nuclear and cytoplasmic fractions of the cells were extracted separately with the CelLytic NuCLEAR Extraction Kit (CAS No.: 2900, Sigma-Aldrich) according to the manufacturer’s instructions. Pim-3 and miR-936 were amplified with SYBR Green (CAS No.: #1708880, Bio-Rad Laboratories). The distribution of NRF2 in the cytoplasm and nucleus was confirmed via Real-time PCR. The relative expression of the indicated genes was ascertained via comparison with the expression of GAPDH or U6. The primers were synthesized and purchased from Sangon Biotech Co.,Ltd. (Shanghai), and were shown in **Supplementary Table S1**.

### Western blot assay

The indicated tissues and cells were dissociated using RIPA lysis buffer (CAS No.: R0020, Solarbio Science & Technology Co., Ltd., Beijing, China). Total protein was extracted and quantified using a BCA Protein Assay Kit (CAS No.: 23227, Thermo Scientific). The protein samples (30 mg/lane) were separated by 12% sodium dodecyl sulfate-polyacrylamide gel electrophoresis (SDS-PAGE) and transferred to nitrocellulose membranes. The membranes containing the target proteins were blocked in Tris-buffered saline with 5% (w/v) non-fat dry milk at 37°C for 6 hours and then incubated with primary antibodies over night at 4°C. Finally, the membranes were incubated with goat Anti-Rabbit IgG H&L (HRP) (1:100 dilution, #ab6721, Abcam) for 1 h at room temperature. Immunoreactive proteins were visualized using the Molecular Imager ChemiDoc^TM^ XRS+ System (Bio-Rad).

### RNA pull-down assay

Huh7/SOR and HepG2/SOR cells were treated with GV657-Pim-3 vector, followed by transfection with biotinylated miR-936 mimics (miR-936 mimics) or MUT (mutant) mimics using Lipofectamine 2000. After 48 h, the cells were lysed, sonicated, and incubated with M-280 streptavidin magnetic beads (CAS No.: 11205D, Invitrogen). The abundance of Pim-3 in the bound fractions was determined via real-time PCR.

### Co-immunoprecipitation and pull-down assay

Cells were collected and lysed on ice. The cell supernatant was incubated with protein G-Sepharose at room temperature for 2 h. Alternatively, HepG2/SOR cells coexpressing Flag-Pim-3 and HA-ANKRD18A were lysed in an NP40-based buffer (Sigma-Aldrich). The cell supernatant was cultured with anti-FLAG or anti-HA beads (Sigma-Aldrich) for another 2 h. Western blot assay was performed using the indicated antibodies. For the pull-down assay, human p3×FLAG-CMV-14-Src, pET302 NT-His-ANKRD18A, and pCMV6-Myc-NRF2 plasmids were purchased from Sangon Biotech Co. Ltd. HEK-293 cells coexpressing Flag-Src, His-ANKRD18A, and Myc-NRF2 were lysed in 1% Triton X-100. Cell supernatants were cultured with the indicated antibodies for 2h at 4°C, followed by treatment with protein G-Sepharose beads (#ab193259, Abcam) for another 2 h. All the beads were washed using AminoLinkPlus Resin and Pierce Control Agarose Resin (CAS No.: 20475, Thermo Scientific) and prepared for Western blot assay.

### mRNA microarray analysis

Total RNA was extracted from the cells using a Cells-to-CT Kit (Thermo Fisher Scientific). RNA integrity was quantified using a UV spectrophotometer (Beckman Coulter, Brea, CA, USA) and Agilent Bioanalyzer 2000 (Agilent). MRNA expression profiles were determined using the Human Clariom S Assay platform (Affymetrix). Briefly, RNA samples were mixed with primers containing a T7 promoter, and reverse transcription was performed to synthesize first-strand cDNA, prior to the synthesis of second-strand cDNA and cRNA. The cRNA was converted to biotinylated double-stranded cDNA (ds-cDNA) hybridization targets for unbiased coverage of the transcriptome using the GeneAtlas® Hybridization Station (Affymetrix). The arrays were stained with an Affymetrix GeneChip Fluidics Station 450 system and scanned with a GeneChip Scanner 3000 7G (Affymetrix). Differentially expressed genes (DEGs) were defined as those with a fold-change ≥1.5 and a p value<0.05. These DEGs were prepared for Kyoto Encyclopedia of Genes and Genomes (KEGG) pathway and Gene Ontology (GO) term enrichment analyses, which were implemented based on the GSEA database using NCBI Gene Expression Omnibus, Bioconductor, and STRING 9.1.

### High-content screening

Huh7/SOR cells were seeded into 48-well plates and transfected with miRNA mimics or inhibitors of miR-33a, miR-936, miR-149a, miR-23a, miR-124-3p.1 and miR-17, or transduced with shRNA plasmids to silence ANKRD18A and SRC genes. The expression of Pim-3 and the indicated mRNAs was confirmed using a Celigo® Image Cytometer and the Cellomics Array Scan System.

### miRNA microarray analysis

Small RNAs from the indicated cells were isolated using an Agilent Bioanalyzer and then purified and recovered on a denaturing acrylamide gel using a Flash PAGE Electrophoresis Kit (Ambion, Thermo Fisher Scientific), according to the manufacturer’s protocol (http://www.ambion.com/techlib/prot/bp_13100;). TRIzol-purified RNA (100 ng) was labeled using Ambion’s mirVANA labeling kit according to the manufacturer’s instructions (http://www.ambion.com/techlib/prot/fm_1562). Labeled RNA was placed in a hybridization chamber (mirVANA miRNA Bioarray Essentials Kit, CAS No.: AM1561, Thermo Fisher Scientific) over arrays containing probes whose sequences were obtained from the Sanger miRbase V18.0 database (http://www.sanger.ac.uk/Software/Rfam/mirna).

The hybridization chambers were washed and scanned according to the guidelines (Agilent Technologies, Santa Clara, CA, USA). The microarray image information was transformed into an intensity value, and the signal was directly output into GeneSpring GX 12.5 software for quartile standardization (Agilent Technologies, Santa Clara, CA). Differentially expressed miRNAs were screened via a paired t test with the cutoff criteria of fold change ≥1.5 and p value<0.05.

### Statistical analysis

Group comparisons were performed using paired Student’s t test and Mann-Whitney U test using the SPSS software (version 17.0; SPSS Inc., Chicago, IL, USA). The level of significance was set at ^*^P< 0.05.

## Results

### Pim-3 induces sorafenib resistance in liver cancer

**Figure 1:**
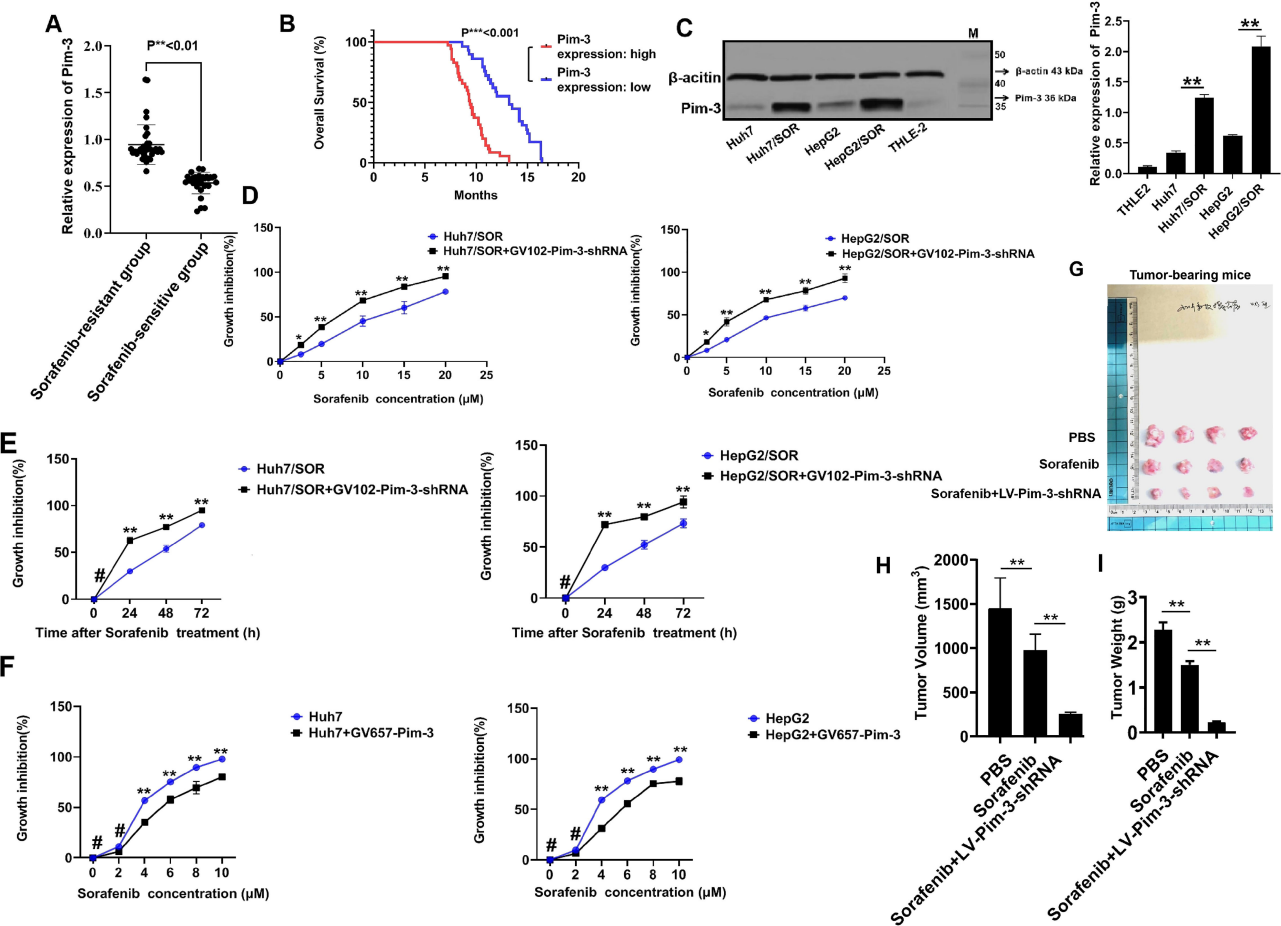
Pim-3 is an initiator of sorafenib resistance in liver cancer. **(A)** Pim-3 expression in liver cancer tissues from sorafenib-sensitive and sorafenib-resistant patients was detected by Western blot assay. Data were shown as the means ± SD from three independent experiments. **P<0.01. **(B)** Kaplan-Meier survival curves based on the correlation between Pim-3 expression and overall survival rate of liver cancer patients were generated using the log-rank test. . **(C)** Pim-3 expression in sorafenib-resistant and parental cells was determined by Western blot assay. The results were shown as the representatives (left) and means ± SD (right) from three irrelevant experiments.^**^P<0.01. **(D, E)** Huh7/SOR and HepG2/SOR cells were pretransfected with the GV102-Pim-3-shRNA vector for 24h and then treated with sorafenib at increasing concentrations (0,2.5, 5, 10, 15 and 20 μM) for another 48 h **(D)** or with sorafenib (10 μM) for 0-72 h **(E)**.The growth inhibition of Huh7/SOR and HepG2/SOR cells was analyzed using CCK-8 assay. **(F)** Huh7 and HepG2 cells were pretransduced with the GV657-Pim-3 plasmid for 24 h, followed by the stimulation with sorafenib at increasing concentrations (0, 2, 4, 6, 8 and 10 µM) for another 48h.The inhibitory effect of sorafenib on cell growth was confirmed with the help of CCK-8 analysis. In **(D–F)**, data were expressed as the means ± SD of three replicates. *P<0.05,**P<0.01 and ^#^P>0.05. **(G–I)** Tumor-bearing mice were administered with LV-Pim-3-shRNA plasmid and then given sorafenib (30 mg/kg). Tumor presentations **(G)**, tumor volume **(H)** and tumor weight **(I)** in each group were demonstrated and examined. The results were demonstrated as the representatives **(G)** and the means ± SD from triplicate experiments **(H, I)**. *P<0.05 and **P<0.01.

To date, the clinical relevance of Pim-3 expression in liver cancer patients undergoing sorafenib therapy remains unclear. Our follow-up of patients who received sorafenib therapy verified that Pim-3 expression in sorafenib-resistant patients was significant higher than that in sorafenib-sensitive patients (**Supplementary Figure S1**; **Figure 1A**). The patients with elevated Pim-3 expression had a poorer prognosis, which was described as a shorter OS time than did the other patients (**Figure 1B**). Western blot assays further confirmed that the Pim-3 protein abundance was greater in Huh7/SOR and HepG2/SOR cells than that in the parental cells (**Figure 1C**). Pim-3 knockdown significantly increased the sensitivity of Huh7/SOR and HepG2/SOR cells to sorafenib (**Figures 1D, E**). However, the cytotoxic activity of sorafenib in Huh7 and HepG2 cells was decreased after Pim-3 overexpression (**Figure 1F**). In addition, tumor-bearing mice treated with a combination of LV-Pim-3-shRNA plasmid and sorafenib showed a larger reduction in tumor burden (**Figure 1G**) and more aggressive decreases in tumor volume and weight than did those treated with PBS and sorafenib monotherapy (**Figures 1H, I**). These findings indicate that Pim-3 may perpetually promote the development of sorafenib resistance in liver cancer.

### MiR-936 targets the 3’-UTR of Pim-3 to suppress its expression in sorafenib-resistant liver cancer cells

**Figure 2:**
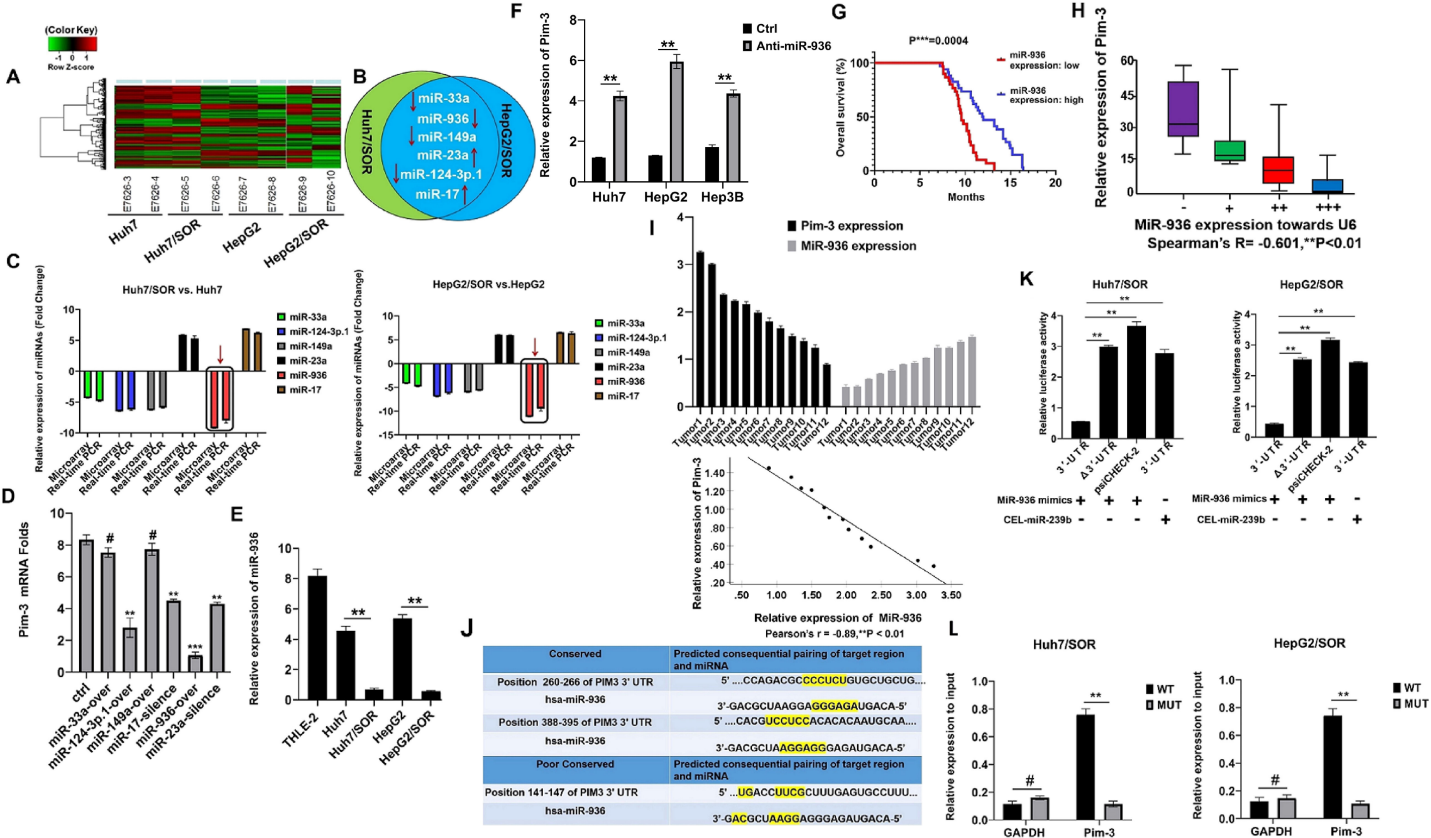
MiR-936 inhibits Pim-3 expression in sorafenib-resistant liver cancer cells by directly targeting its 3′-UTR. **(A)** A heatmap of hierarchical clustering showing the differentially expressed miRNAs in Huh7/SOR vs. Huh7 and HepG2/SOR vs. HepG2 cells. **(B)** The venn diagram depicting six miRNAs differentially expressed in both Huh7/SOR and HepG2/SOR cells, compared with Huh7 and HepG2 cells, respectively. **(C)** The expression profiles of the differentially expressed miRNAs were identified using microarray assay and subsequent real-time PCR validation. **(D)** Huh7/SOR cells were seeded into 48-well plates and transfected with indicated miRNA mimics and inhibitors. Pim-3 mRNA was detected by HCS with the assistance of Celigo® Image Cytometer and Cellomics Array Scan System. **(E)** MiR-936 expression in sorafenib-resistant and parental cells was measured via real-time PCR assay. **(F)** Pim-3 expression in liver cancer cells with or without miR-936 inhibitor (anti-miR-936) transfection was detected using real-time PCR assay. **(G)** Kaplan-Meier OS curves for liver cancer patients based on the miR-936 expression level were generated using the log-rank test. P***<0.001. **(H)** Correlation between Pim-3 and miR-936 expression in tumor tissues from primary liver cancer patients. (Spearman’s R=-0.601, ^**^P<0.01). **(I)** Pim-3 and miR-936 mRNA levels were detected. *Pearson correlation analysis* between Pim-3 and miR-936 mRNA levels in tumor tissues from liver cancer-bearing mice was performed. (Pearson’s r = -0.89, **P < 0.01). **(J)** MiR-936 and the 3′-UTR sequences of Pim-3 were complementary to each other, according to the predictions made with the TargetScan algorithm. **(K)** Huh7/SOR and HepG2/SOR cells were cotransfected with the reporter construct and miR-936 mimics and cel-miR-239b. The Renilla luciferase activity of reporter constructs (psiCHECK-2) containing the wild-type and mutated Pim-3 3′-UTR was evaluated 48 h after transfection. **(L)** Huh7/SOR and HepG2/SOR cells were cotransfected with GV657-Pim-3 vector and biotinylated miR-936 mimics or its MUT mimics. The abundance of Pim-3 in the bound fractions was checked using Real-time PCR assay. In **(D-F, K, L)**, the data were representative of three independent experiments with means ± SD. ^**^P<0.01 and ^#^P>0.05.

Previously, microRNAs (miRNAs) have been reported to modulate the expression of Pim-1 and Pim-2 in HCC cells (35, 36). To explore whether Pim-3 expression can be modulated by miRNAs in liver cancer cells, the AgilentGeneChip^®^ was used to identify differentially expressed miRNAs (**Figure 2A**). The miRNAs with differentially expression in the top ten in Huh7/SOR and HepG2/SOR cells compared to their respective parental cells were screened. Six miRNAs with differential expression in both Huh7/SOR and HepG2/SOR cells were confirmed (**Figure 2B**). The results of both the miRNA microarray assay and subsequent real-time PCR validation demonstrated that miR-936 was the most downregulated miRNA in Huh7/SOR and HepG2/SOR cells (**Figure 2C**).

Next, the effects of silencing or overexpressing the selected miRNAs on Pim-3 expression were ascertained via HCS. As shown in **Figure 2D**, Pim-3 mRNA was decreased with most significant change after miR-936 overexpression. The results of real-time PCR assay confirmed that miR-936 expression in both Huh7/SOR and HepG2/SOR cells was lower than that in the corresponding parental cells (**Figure 2E**). The miR-936 inhibitor significantly increased Pim-3 expression in Huh7 and HepG2 cells (**Figure 2F**). Furthermore, miR-936 expression was negatively correlated with OS in liver cancer patients (**Figure 2G**). There was also a negative correlation between Pim-3 and miR-936 expression in liver cancer tissues (**Figures 2H, I**). Additionally, analysis of the miRNA-binding sites in Pim-3 mRNA was performed via an online prediction approach using the chimeric algorithm, followed by the dual luciferase reporter assay. MiR-936 bound to a highly conserved sequence in the 3’-UTR of Pim-3 (**Figure 2J**) and cotransfection with miR-936 mimics, but not cel-miR-239b mimics, repressed the luciferase activity of the wild-type Pim-3 3’-UTR construct (but not that of the mutant Pim-3 Δ3’-UTR or the psiCHECK-2 empty vector) (**Figure 2K**). Moreover, miR-936 mimic-WT captured more Pim-3 than its MUT mimics did (**Figure 2L**). Therefore, we can infer that miR-936 is located upstream of Pim-3 and downregulates Pim-3 expression by directly targeting its 3’-UTR.

### Upregulation of Pim-3 mediated by low miR-936 expression activates the ANKRD18A/Src/NRF2 pathway

**Figure 3:**
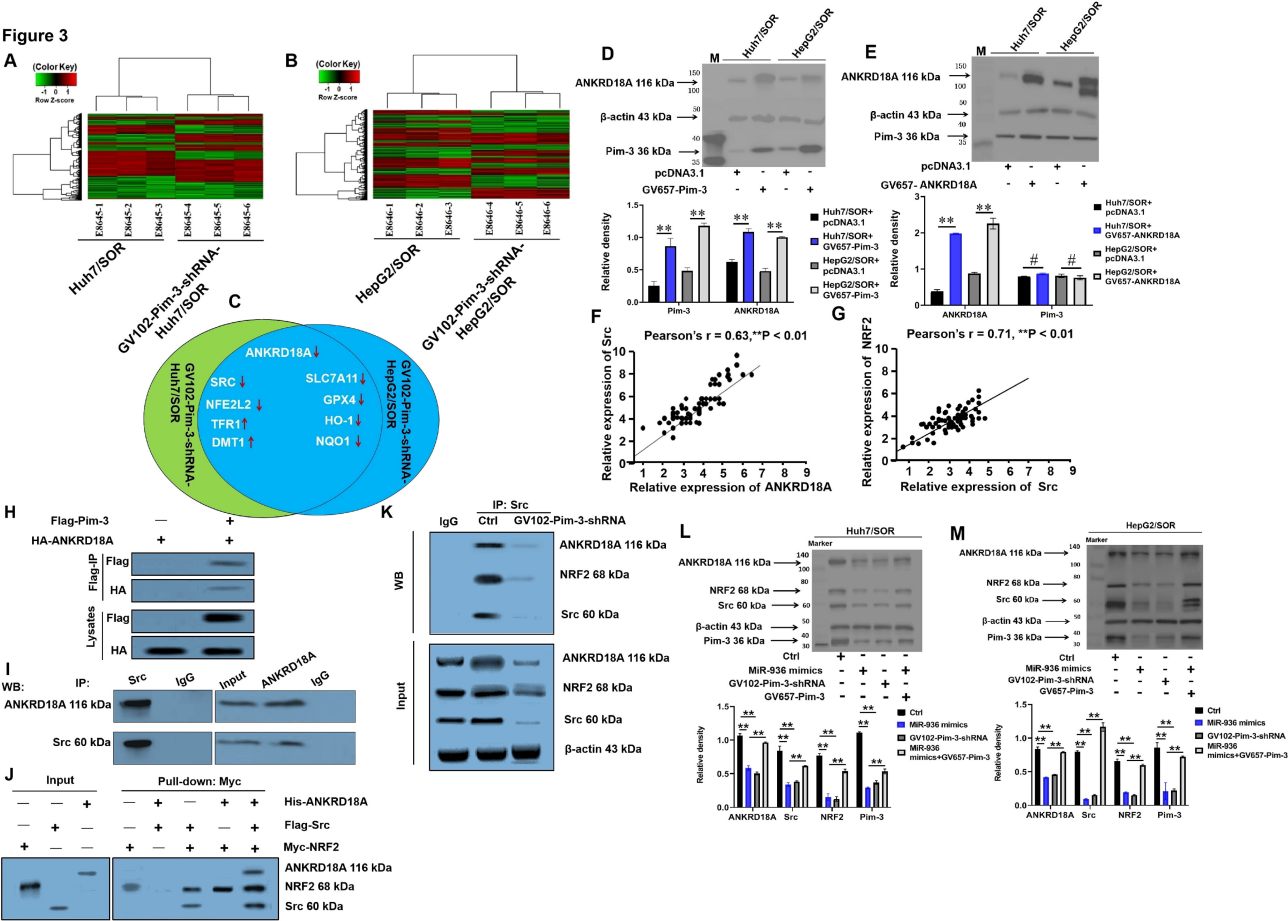
Low miR-936-induced upregulation of Pim-3 activates the ANKRD18A/Src/NRF2 pathway. **(A, B)** Heatmaps showing differentially expressed genes in GV102-Pim-3-shRNA-Huh7/SOR *vs.* Huh7/SOR **(A)** and GV102-Pim-3-shRNA-HepG2/SOR *vs.* HepG2/SOR cells **(B)** generated using an R package. **(C)** Transcripts with a fold change≥3 in mRNA expression in GV102-Pim-3-shRNA-Huh7/SOR and GV102-Pim-3-shRNA-HepG2/SOR relative to Huh7/SOR and HepG2/SOR cells, respectively. **(D)** The effect of Pim-3 overexpression on the ANKRD18A protein level was assessed using Western blot assay. **(E)** The effect of ANKRD18A overexpression on the Pim-3 protein level was evaluated by Western blot assay. **(F, G)** The correlations between Src and ANKRD18A **(F)**, or between NRF2 and Src protein expression **(G)** were determined via *Pearson correlation analysis*. **(H, I)** Co-IP assay using anti-Flag or anti-HA **(H)**, and anti-ANKRD18A or anti-Src **(I)** antibodies were performed in HepG2/SOR cells. **(J)** Pull-down assay using anti-His, anti-Flag or anti-Myc antibodies was executed in HEK-293 cells that were transfected with a combination of the p3×FLAG-CMV-14-Src, pET302 NT-His-ANKRD18A and pCMV6-Myc-NRF2 plasmids. **(K)** HepG2/SOR cells were pretransfected with GV102-Pim-3-shRNA plasmid for 24h. Co-IP assay was carried out to assess the interactions between ANKRD18A and Src, or between Src and NRF2 using anti-ANKRD18A, anti-Src and anti-NRF2 antibodies. **(L, M)** Huh7/SOR **(L)** and HepG2/SOR **(M)** cells were pretreated with the miR-936 mimics, the GV102-Pim-3-shRNA plasmid, or the combination of miR-936 mimics and GV657-Pim-3 plasmid for 24 h. Western blot assay was used to identify the expression of ANKRD18A, Src and NRF2.For the Western blot assay, the data were represented as the representatives images (upper), as well as the means ± SD of the relative intensities that were normalized to that of β-actin (lower). ^**^P<0.01 and ^#^P>0.05.

To elucidate the mechanisms by which the miR-936/Pim-3 axis inhibits sorafenib resistance in liver cancer, the mRNA expression profiles of GV102-Pim-3-shRNA-Huh7/SOR *vs.* Huh7/SOR, and GV102-Pim-3-shRNA-HepG2/SOR *vs.* HepG2/SOR cells were analyzed using the Affymetrix Clariom^TM^ S GeneChip (**Figures 3A, B**). Two upregulated including TFR1 and DMT1; 7 downregulated clusters including ANKRD18A, SRC, NFE2L2, SLC7A11, GPX4, NQO1 and HO-1, were identified after Pim-3 gene was silenced (**Figure 3C**). KEGG pathway and GO term enrichment analyses further confirmed that these DEGs were enriched in the *Nrf2* pathway (**Supplementary Figure S2**) and encoded ankyrin repeat domain-containing protein 18A (ANKRD18A), the proto-oncogene tyrosine-protein kinase Src, NRF2, and prototypical proteins involved in the modulation of iron homeostasis and lipid peroxidation, including SLC7A11, GPX4, NQO1, TFR1, HO-1, and DMT1(**Supplementary Figures S3**, **S4**). Among these nine transcripts, ANKRD18A was defined as having the most obvious reduction in mRNA expression (**Supplementary Figure S4**), and Pim-3 unidirectionally promoted the expression of the ANKRD18A protein (**Figures 3D, E**). Interestingly, the SRC and NFE2L2 mRNA levels showed the most significant decrease after gene silence of ANKRD18A and SRC, respectively (**Supplementary Figures S5**, **S6**). As displayed in **Figures 3F, G**, positive correlations were observed between Src and ANKRD18A expression and between NRF2 and Src expression in liver cancer tissues. These results indicate that SRC and NFE2L2 were both nonnegligible components located downstream of ANKRD18A and that the ANKRD18A/Src/NRF2 axis also arrived at a historic moment. Co-IP and pull-down assays further elaborated that the Pim-3 and ANKRD18A, as well as the ANKRD18A and Src proteins, were directly colocalized in HepG2/SOR cells (**Figures 3H, I**); however, His-ANKRD18A was captured only by Myc-NRF2 in the presence of Flag-Src, indicating an indirect and Src-dependent interaction between ANKRD18A and NRF2 (**Figure 3J**). Strikingly, the direct interactions between endogenous ANKRD18A and Src and between Src and NRF2 were noticeably attenuated following transfection with miR-936 mimics and GV102-Pim-3-shRNA plasmid (**Figure 3K**). Additionally, introduction of the miR-936 mimics and GV102-Pim-3-shRNA plasmid substantially downregulated the protein levels of ANKRD18A, Src, and NRF2. However, Pim-3 overexpression partially restored the expression of ANKRD18A, Src, and NRF2, which was reduced by the miR-936 mimics (**Figures 3L, M**). In general, the overwhelming expression of Pim-3 precipitated by a low miR-936 level plays an active role in guiding the activation of the ANKRD18A/Src/NRF2 pathway, in which the expression of indicated proteins related to iron homeostasis and lipid peroxidation is rearranged to favor ferroptosis in liver cancer.

### Upregulation of Pim-3 induced by low miR-936 expression protects sorafenib-resistant liver cancer cells against ferroptosis by activating the ANKRD18A/Src/NRF2 pathway

**Figure 4:**
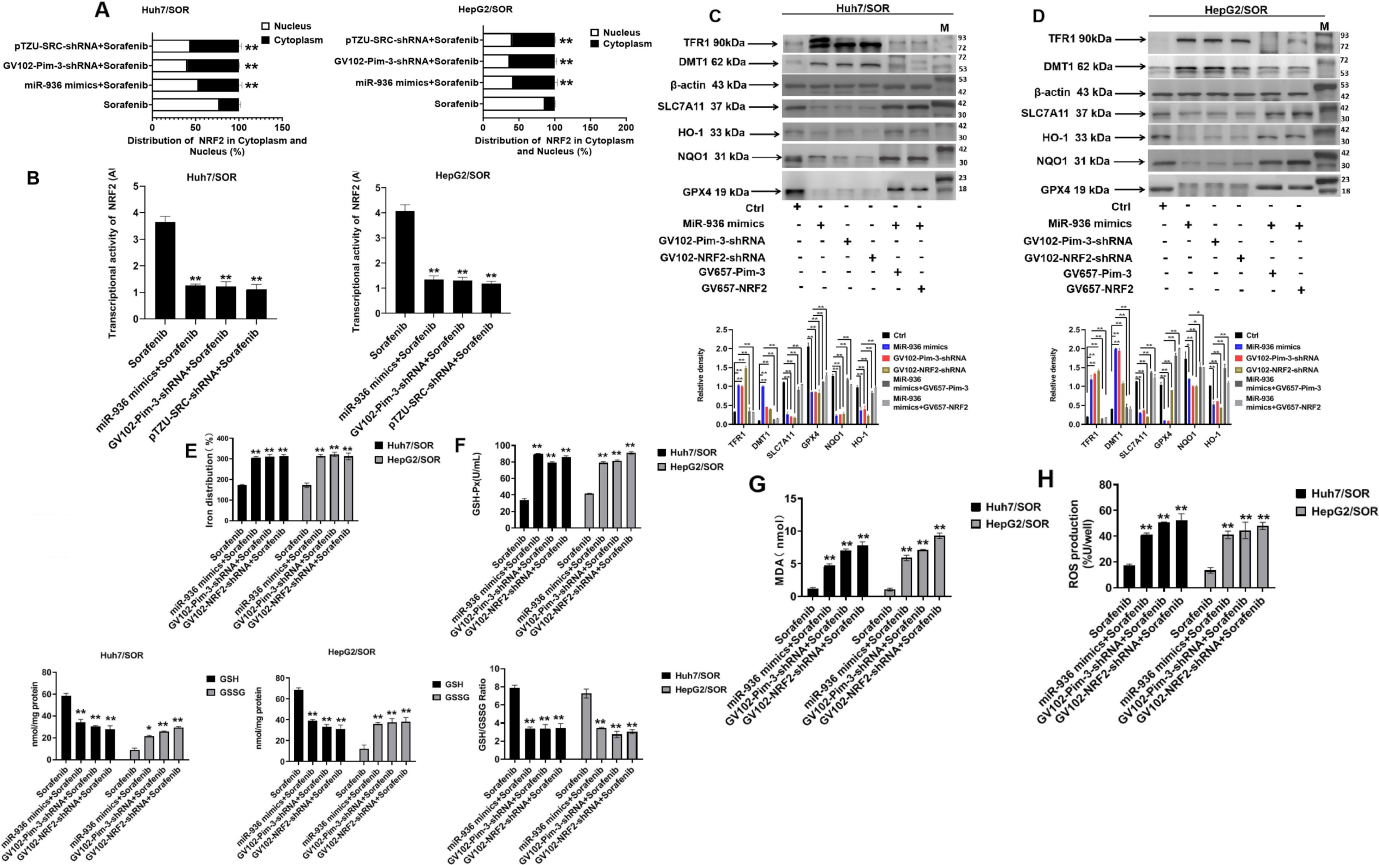
Low miR-936-induced upregulation of Pim-3 represses ferroptosis in liver cancer by activating the ANKRD18A/Src/NRF2 pathway. **(A)** Huh7/SOR and HepG2/SOR cells were treated with miR-936 mimics, GV102-Pim-3-shRNA plasmid, and pTZU-SRC-shRNA plasmid for 24 h prior to treatment with sorafenib (10 μM) for another 48 h. The distribution of NRF2 in the cytoplasm and nucleus was confirmed using real-time PCR assay. **(B)** Huh7/SOR and HepG2/SOR cells were treated with miR-936 mimics, GV102-Pim-3-shRNA plasmid, and pTZU-SRC-shRNA plasmid for 24h, followed by the stimulation with sorafenib (10μM) for another 48 h. NRF2 transcription activity was confirmed using *chorion specific transcription factor NRF*2 assay. **(C, D)** Huh7/SOR and HepG2/SOR cells were pretransfected with miR-936 mimics, GV102-Pim-3-shRNA plasmid, GV102-NRF2-shRNA plasmid, the combination of miR-936 mimics and GV657-Pim-3 plasmid, or the combination of miR-936 mimics and GV657-NRF2 plasmid for 24 h, and then stimulated with sorafenib (10 μM) for another 48 h. Western blot assay was used to examine the protein level of TFR1, DMT1, SLC7A11, GPX4, NQO1, and HO-1. **(E–H)** Huh7/SOR and HepG2/SOR cells were transfected with miR-936 mimics, GV102-Pim-3-shRNA plasmid and GV102-NRF2-shRNA plasmid for 24 h, and then managed with sorafenib (10 μM) for another 48 h. Iron distribution **(E)**, GSH level **(F)**, lipid peroxide level **(G)** and ROS level **(H)** were judged using the Iron Assay, GSH/GSSG and GSH-px standard quantitative assay, Lipid Peroxidation (MDA) Assay and DCFH-DA Staining Assay Kit, respectively. The results were shown as the means ± SD from triplicate experiments. ^**^P<0.01 and ^*^P<0.05.

The role of increased NRF2 transcriptional activity in negatively governing ferroptosis sensitivity by upregulating the expression of antioxidants and increasing the iron pool has been well documented (20–25). Here, NRF2 was found to be redistributed in the nuclear and cytoplasmic fractions, and transduction of the GV102-Pim-3-shRNA and pTZU-SRC-shRNA plasmids prevented NRF2 from entering the nucleus in Huh7/SOR and HepG2/SOR cells (**Figure 4A**). Moreover, pretransfection with miR-936 mimics, GV102-Pim-3-shRNA or pTZU-SRC-shRNA plasmid distinctly inhibited the transcriptional activity of NRF2 in Huh7/SOR and HepG2/SOR cells (**Figure 4B**).

Accordingly, we hypothesized that aberrant expression of Pim-3 driven by a low miR-936 level activates the ANKRD18A/Src/NRF2 pathway to enhance the transcriptional activity of NRF2, thereby regulating iron homeostasis and lipid peroxidation and constraining sorafenib-induced ferroptosis in liver cancer cells. As expected, overexpression of miR-936 and knockdown of Pim-3 and NRF2 dramatically increased the protein levels of TFR1 and DMT1 but decreased those of SLC7A11, GPX4, NQO1, and HO-1 in Huh7/SOR and HepG2/SOR cells. However, Pim-3 and NRF2 overexpression reversed the effects of miR-936 mimic transfection on the expression of these ferroptosis-related markers (**Figures 4C, D**). In addition, the upregulation of Fe^2+^, lipid peroxides and ROS levels, as well as GSH reduction were confirmed in Huh7/SOR and HepG2/SOR cells treated with miR-936 mimics, GV102-Pim-3-shRNA plasmid and GV102-NRF2-shRNA plasmid (**Figures 4E–H**). Therefore, Pim-3, whose expression was positively correlated with low a miR-936 level, suppressed ferroptosis in sorafenib-resistant liver cancer cells by accelerating the activation of the ANKRD18A/Src/NRF2 pathway.

### Upregulation of Pim-3 induced by low miR-936 expression fuels sorafenib resistance in liver cancer by impeding cell ferroptosis

**Figure 5:**
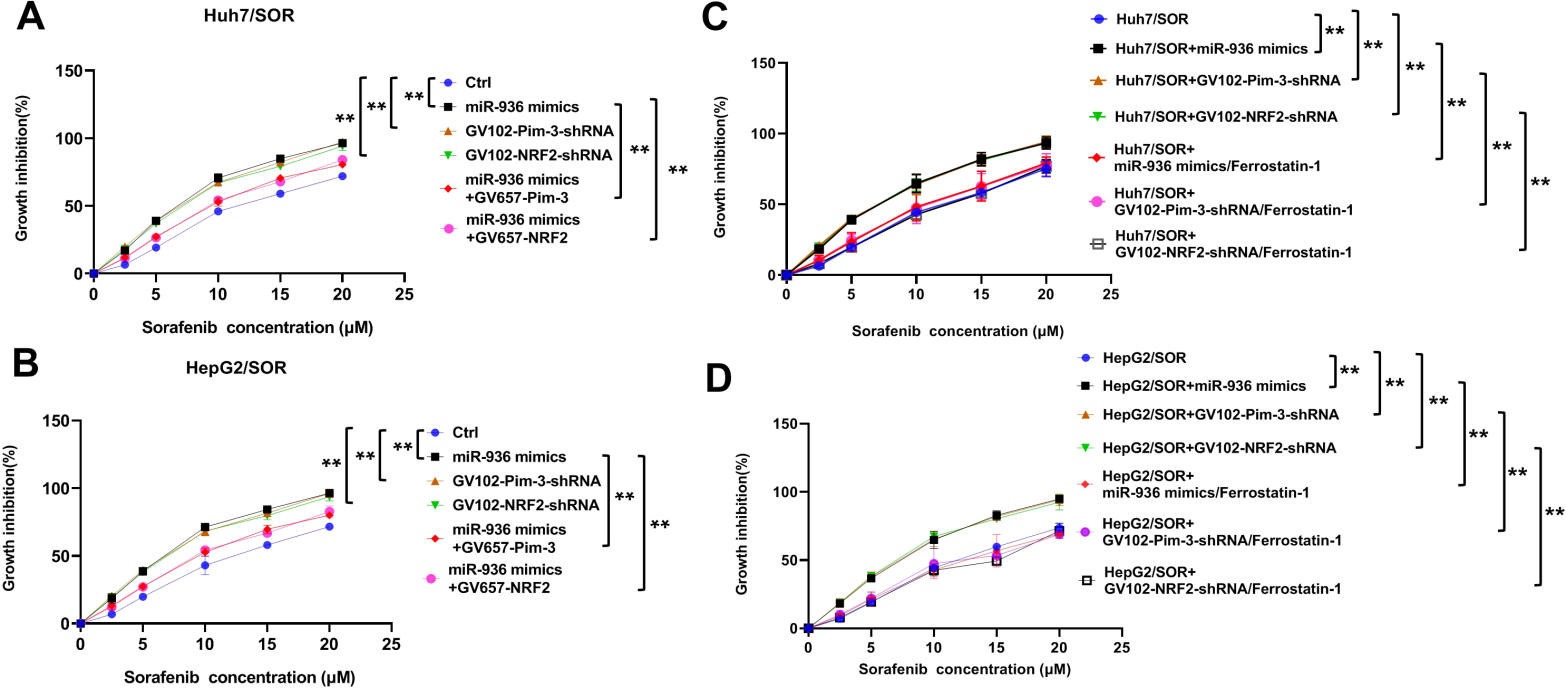
Low miR-936-induced upregualtion of Pim-3 mediates sorafenib resistance in liver cancer by the induction of ferroptosis blockade. **(A, B)** Huh7/SOR and HepG2/SOR cells were pretransfected with miR-936 mimics, GV102-Pim-3-shRNA plasmid, GV102-NRF2-shRNA plasmid, the combination of miR-936 mimics and GV657-Pim-3 plasmid, or the combination of miR-936 mimics and GV657-NRF2 plasmid for 24 h. These cells were then incubated with sorafenib (0, 2.5, 5, 10, 15 and 20 μM) for another 48h. The growth inhibition of indicated cells was determined using CCK-8 method. **(C, D)** Huh7/SOR and HepG2/SOR cells were pretransfected with miR-936 mimics, GV102-Pim-3-shRNA plasmid and GV102-NRF2-shRNA plasmid, or the combination of indicated plasmids and ferrostatin-1 (2 μM) for 24 h prior to incubation with sorafenib (0, 2.5, 5, 10, 15 and 20 μM) for another 48h. The growth inhibition of indicated cells were ascertained using CCK-8 method. In **(A–D)**, the results were expressed as the means ± SD of three replicates. ^**^P<0.01.

Next, we aimed to determine whether extreme Pim-3 expression induced by a low miR-936 level can mediate sorafenib resistance in liver cancer. As depicted in **Figures 5A, B**, introduction of miR-936 mimics, GV102-Pim-3-shRNA plasmid and GV102-NRF2-shRNA plasmid increased the sensitivity of Huh7/SOR and HepG2/SOR cells to sorafenib. However, the sorafenib-sensitization effect of miR-936 mimics was partially blocked by Pim-3 and NRF2 overexpression (**Figures 5A, B**).

More importantly, the enhancement in sorafenib sensitivity driven by the introduction of miR-936 mimics, GV102-Pim-3-shRNA or GV102-NRF2-shRNA plasmid, was abolished by treatment with ferrostatin-1, a well-known inhibitor of ferroptosis (**Figures 5C, D**).

In summary, we believe that overexpression of Pim-3 upregulation caused by underexpression of miR-936 promotes t sorafenib resistance in liver cancer, partially by inhibiting ferroptosis.

## Discussion

Liver cancer is one of the most commonly diagnosed cancers in humans, characterized by insidious onset, rapid progression, and a high degree of malignancy (37). Despite the continuous development of new strategies such as radiochemotherapy, radiofrequency ablation, immunotherapy, and even liver transplantation, advanced liver cancer is difficult to manage clinically and is rarely successfully treated (38, 39). Sorafenib is the first approved systemic therapy for advanced liver cancer but has been shown to offer unfavorable outcomes, especially in patients who develop resistance to the drug (40). However, the mechanisms underlying the progression of sorafenib resistance in liver cancer that were closely related to the obstruction of ferroptosis, are not fully understood.

Our previous study raised the possibility that Pim-3 expression is positively linked to the occurrence of multidrug resistance (MDR) in HCC. In particular, Pim-3 depletion stimulated cisplatin-induced apoptosis by restricting the expression of BCL-2, BCL-XL and p-Bad, and increased the intracellular drug concentration by reducing the expression of MDR-associated proteins, thereby reversing MDR in HCC (34). Still, the role of Pim-3 in promoting sorafenib resistance remains us lots of to elucidate. In the present study, it was demonstrated for the first time that Pim-3 can promote sorafenib resistance in liver cancer. As reported previously, miR-33a and miR-574-3p were shown to downregulate Pim-3 expression in human colorectal and pancreatic cancer cells, respectively (41, 42). Herein, our findings corroborated that miR-936 directly targets the 3’-UTR of Pim-3 to decrease its expression and thus was not only the first of its kind but also complementary to its predecessors.

To our knowledge, no reports have mentioned the signaling pathways activated by miR-936/Pim-3, and little is known about the precise mechanisms underlying the progression of sorafenib resistance triggered by miR-936/Pim-3 in liver cancer. Here, credible targets involved in Pim-3-induced sorafenib resistance in liver cancer were identified with mRNA microarray analysis. To our knowledge, ours is the first study to report the regulatory effects of miR-936/Pim-3 on ANKRD18A and Src expression, and the existence of the miR-936/Pim-3-ANKRD18A/Src/NRF2 axis. Transfection with miR-936 mimics and GV102-Pim-3-shRNA plasmid interfered with the interactions between the ANKRD18A and Src proteins and between the Src and NRF2 proteins, also reducing ANKRD18A, Src, and NRF2 expression. Introduction of the miR-936 mimics, GV102-Pim-3-shRNA plasmid, and pTZU-SRC-shRNA plasmid also impeded the entry of NRF2 into the nucleus and suppressed its transcriptional activity. Considering the above findings, the upregulation of Pim-3 which is driven predominantly by a low miR-936 level, can activate the ANKRD18A/Src/NRF2 pathway, probably in turn enhancing NRF2 transcriptional activity and causing sorafenib resistance in liver cancer.

As a major gatekeeper of intracellular redox balance, NRF2 is a master regulator of the tumor cell response to ROS (43). The only regulatory factors correlated with NRF2 activation were initially thought to be p62 and Kelch-like ECH-associated protein 1 (Keap1) (27, 44), although AMPK and P53 also contribute to arbitrating the NRF2 antioxidant defense system during hypoxia/reoxygenation (45, 46). NRF2 dissociates from Keap1 via autoubiquitination-mediated degradation and is quickly translocated into the nucleus to form a heterodimer with its binding partners, i.e., small v-maf avian musculoaponeurotic fibrosarcoma oncogene homolog (Maf) proteins such as MafG (47, 48). Herein, we lay the foundation for a renewed understanding of NRF2 activation. In particular, miR-936-induced upregulation of Pim-3 increased the protein levels of ANKRD18A, Src, and NRF2. Interestingly, the transcriptional activity of NRF2 was enhanced, benefiting directly from activation of the ANKRD18A/Src/NRF2 pathway. The present data provide specific guidance indicating that Src activation is encouraged by the miR-936/Pim-3/ANKRD18A signaling cascades that promote the nuclear retention as well as the transcriptional activity of NRF2. This hypothesis suggests that ANKRD18A and Src are important elements related to NRF2 activation. Especially, Src upregulation induced the pseudoubiquitination of NRF2 that can increase its nuclear translocation and transcriptional activity. However, further confirmation of the mechanism through which ANKRD18A and Src activate NRF2 is needed.

In the present study, mRNA microarray analysis confirmed that Pim-3 silencing affected the mRNA levels of SLC7A11, GPX4, NQO1, TFR1, HO-1, and DMT1. Thus, the high expression of Pim-3, which is caused by a low miR-936 level, promoted the transcriptional activity of NRF2, rearranging the expression of regulatory proteins related to iron homeostasis and lipid peroxidation. In addition, it is reasonable to conclude that Pim-3 upregulation mediated by a low miR-936 expression promotes sorafenib resistance in liver cancer by inhibiting ferroptosis, in which the activation of the ANKRD18A/Src/NRF2 pathway, especially the transcriptional activity of NRF2, is essential. As expected, transfection with miR-936 mimics, GV102-Pim-3-shRNA plasmid, and GV102-NRF2-shRNA plasmid induced ferroptosis, which was followed by an augmented response to sorafenib, consistent with the hypothesis stated at the beginning of this study. To the best of our knowledge, our study is the first to show that NRF2, in cooperation with the central miR-936/Pim-3/ANKRD18A/Src axis, can shut down new start-up programs of ferroptosis in liver cancer, with increases in TFR1 and DMT1 expression, but decreases in SLC7A11, GPX4, NQO1, and HO-1 protein levels. In summary, NRF2 activation caused by the energizing of the miR-936/Pim-3/ANKRD18A/Src axis in addition to the p62/Keap1/NRF2 pathway constitutes a reliable strategy to escape sorafenib-induced ferroptosis and is ideal for meeting the inordinate demands of liver cancer cells for the acquisition of resistance to sorafenib.

Although abundant evidence suggests that sorafenib acts primarily by sensitizing liver cancer cells to ferroptosis (9–13), a conflicting evidence indicates that sorafenib fails to trigger ferroptosis across a wide range of cancer cell lines (49). Sorafenib has been repeatedly reported to induce ferroptosis through the inhibition of system Xc–, contradicting the claim from the latter study that sorafenib does not trigger ferroptosis through the inhibition of system Xc− due to low SLC7A11 expression in human hepatoma cell lines. Our conclusion is also consistent with these findings, as the characteristics of the sorafenib-resistant liver cancer cells used in our study, such as SLC7A11 expression, may have changed. Interference with the miR-936/Pim-3/ANKRD18A/Src/NRF2 axis increased the protein levels of TFR1 and DMT1 but decreased those of SLC7A11, GPX4, NQO1, and HO-1. Therefore, our findings suggest that “one-solution” reprogramming of ferroptosis, including iron homeostasis and lipid peroxidation, is involved in sorafenib resistance in liver cancer. We will continue to explore whether some yet unrecognized ferroptosis-related mechanisms are triggered when sorafenib is used in liver cancer cells and patients. The key nodes in the miR-936/Pim-3/ANKRD18A/Src/NRF2 axis that regulate ferroptosis resistance in liver cancer should also be further determined. In addition, the exploration of new mechanisms involved in sorafenib resistance that was closely related to Pim-3 should continue to move forward. Actually, Pim-3 has been already shown to have a role in sustaining hepatocarcinogenesis as effector of lysophosphatidic acid receptor 6 (LPAR6) (50). Interestingly, it has been reported that LPAR6 was also associated to sorafenib resistance by promoting lactic acid fermentation at the expense of oxidative phosphorylation (51). Thus, LPAR6/Pim-3-mediated metabolic mechanism encourages sorafenib resistance in HCC and proposes a pharmacological approach to overcome it. These reports and our findings provide us with future research directions. In particular, the role of the miR-936/Pim-3/ANKRD18A/Src/NRF2 axis in promoting sorafenib resistance through LPAR6-mediated metabolic mechanism should be further clarified.

**Figure 6:**
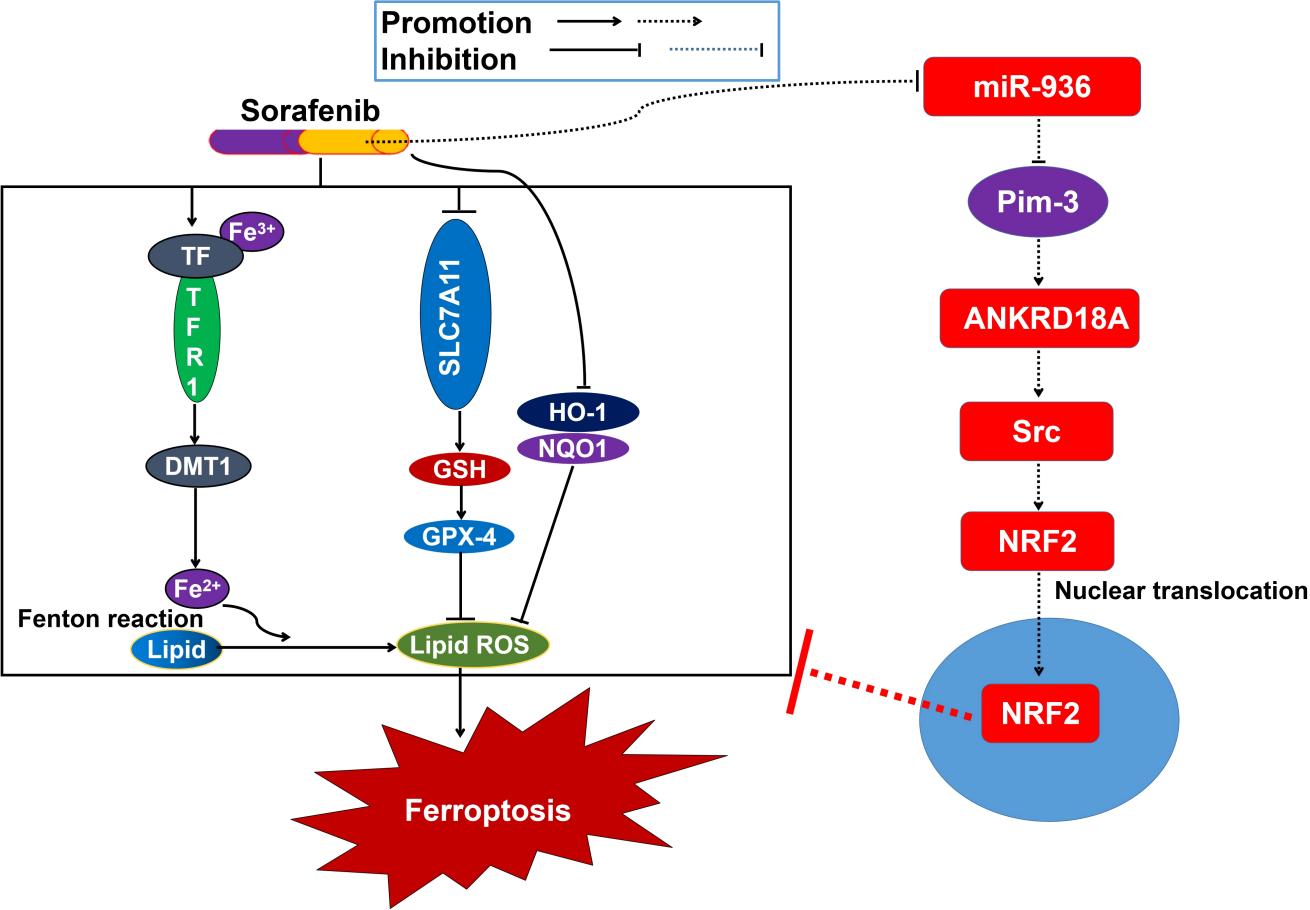
Schematic diagram showing the underlying mechanisms by which Pim-3 meidates sorafenib resistance in liver cancer.

In conclusion, aberrant expression of Pim-3 caused by miR-936 depletion activated the ANKRD18A/Src/NRF2 pathway and promoted the entry of NRF2 into the nucleus. The increased transcriptional activity of NRF2 encourages the reprogramming of iron homeostasis and lipid peroxidation, thus suppressing cell ferroptosis and promoting sorafenib resistance in liver cancer (**Figure 6**). Accordingly, poor responses to sorafenib may be overcome by inhibiting the activation of the ANKRD18A/Src/NRF2 pathway to induce cell ferroptosis, at least partially via miR-936 overexpression and Pim-3 knockdown. Our data open new avenues for the optimization of sorafenib application and highlight the promise of overcoming sorafenib resistance in liver cancer.

## Data Availability

The original contributions presented in the study are included in the article/**Supplementary Material**. Further inquiries can be directed to the corresponding author.

## Supplementary material

Supplementary Figure 1 Representative WB results for Pim-3 in sorafenib-resistant and sorafenib-sensitive patients.

Supplementary Figure 2 KEGG pathway enrichment analysis for these DEGs.

Supplementary Figure 3 GO functional analyses for these DEGs were performed based on the GSEA database.

Supplementary Figure 4 The expression fold changes and encoded proteins of the indicated DEGs were presented.

Supplementary Figure 5 Huh7/SOR cells were transfected with plasmids containing shRNAs targeting the *ANKRD18A* gene. The mRNA expression levels of SRC, NFE2L2, SLC7A11, GPX4, HO-1, NQO1, TFR1 and DMT1 were determined via HCS. The experiments were repeated three times, and the representative results are expressed as the means ± SD. ^**^P<0.01 versus the control.

Supplementary Figure 6 Huh7/SOR cells were transfected with plasmids containing shRNAs targeting the *SRC* gene. The mRNA expression levels of NFE2L2, SLC7A11, GPX4, NQO1, TFR1, HO-1, DMT1 and ANKRD18A were determined via HCS. The experiments were repeated three times, and the representative results are expressed as the means ± SD. ^**^P<0.01 versus the control.
