# Supplementary material for: Low miR-936-mediated upregulation of Pim-3 drives sorafenib resistance in liver cancer through ferroptosis inhibition by activating the ANKRD18A/Src/NRF2 pathway
Source: Front Oncol. 2024 Oct 24;14:1483660. doi: 10.3389/fonc.2024.1483660 (PMC11540556; doi:10.3389/fonc.2024.1483660)
Supplement: Supplementary file 1 [file Table1.docx]

**Table S1 The sequences of Primers used for PCR analysis**

| **Target genes** | **Sequences** |
| --- | --- |
| miR-33a (human ) | Forward: 5'- GGTGCATTGTAGTTGCATTGC-3’  Reverse: 5'-GCGACGAGCAAAAAGCTTGT-3’ |
| miR-149a (human ) | Forward: 5’-GGCTCTGGCTCCGTGTCTT-3’  Reverse: 5’-CTCTGGCTCCGTGTCTTCAC- 3’ |
| miR-23a (human) | Forward: 5’-GCGATCACATTGCCAGGG-3’  Reverse: 5’-AGTGCAGGGTCCGAGGTATT-3’ |
| miR-124-3p.1(human ) | Forward:5’-ACACTCCAGCTGGGTAAGGCACGCGGTG-3’  Reverse: 5’-TGGTGTCGTGGAGTCG-3’ |
| miR-17 (human ) | Forward: 5’-TGCGCCAAAGTGCTTACAGTGCA-3’  Reverse: 5’-CCAGTGCAGGGTCCGAGGTATT-3’ |
| miR-936 (human) | Forward: 5’-CAGACAGTAGAGGGAGGAATC-3’  Reverse: 5′-ACAGTAGAGGGAGGAATCGCAG-3’ |
| U6 | Forward: 5′- CTCGCTTCGGCAGCACA-3’  Reverse: 5’-AACGCTTCACGAATTTGCGT-3’ |
| GAPDH (human ) | Forward: 5’-GAAGGTGAAGGTCGGAGT-3’  Reverse: 5’-CATGGGTGGAATCATATTGGAA-3’ |
| Pim-3(human ) | Forward: 5’-AAGGACGAAAATCTGCTTGTGG-3’  Reverse: 5’-CGAAGTCGGTGTAGACCGTG-3’ |
