# Supplementary figures and images for: Low miR-936-mediated upregulation of Pim-3 drives sorafenib resistance in liver cancer through ferroptosis inhibition by activating the ANKRD18A/Src/NRF2 pathway
Source: Front Oncol. 2024 Oct 24;14:1483660. doi: 10.3389/fonc.2024.1483660 (PMC11540556; doi:10.3389/fonc.2024.1483660)

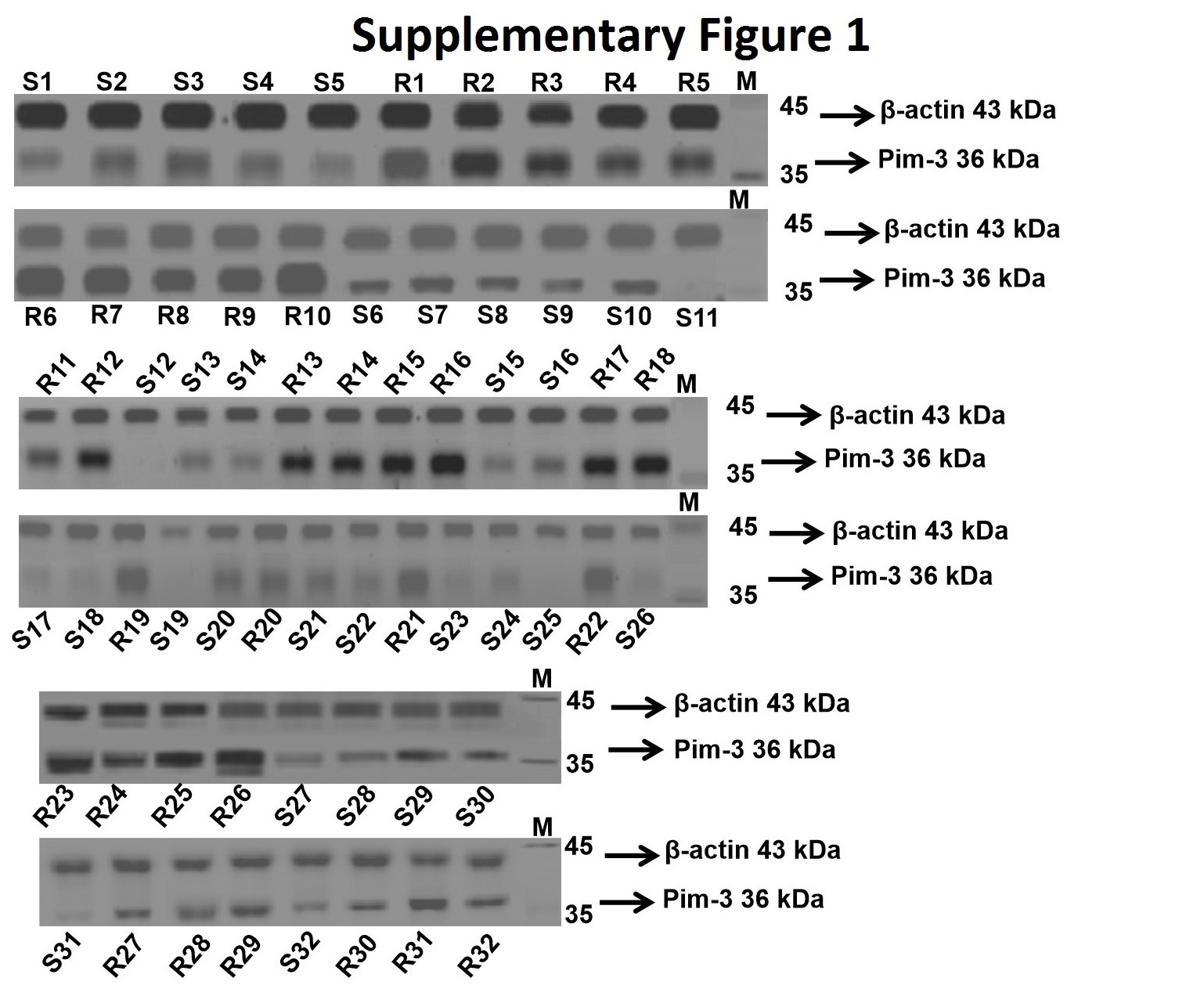

Supplement: Supplementary Figure 1 — Representative WB results for Pim-3 in sorafenib-resistant and sorafenib-sensitive patients. [file Image1.jpeg]

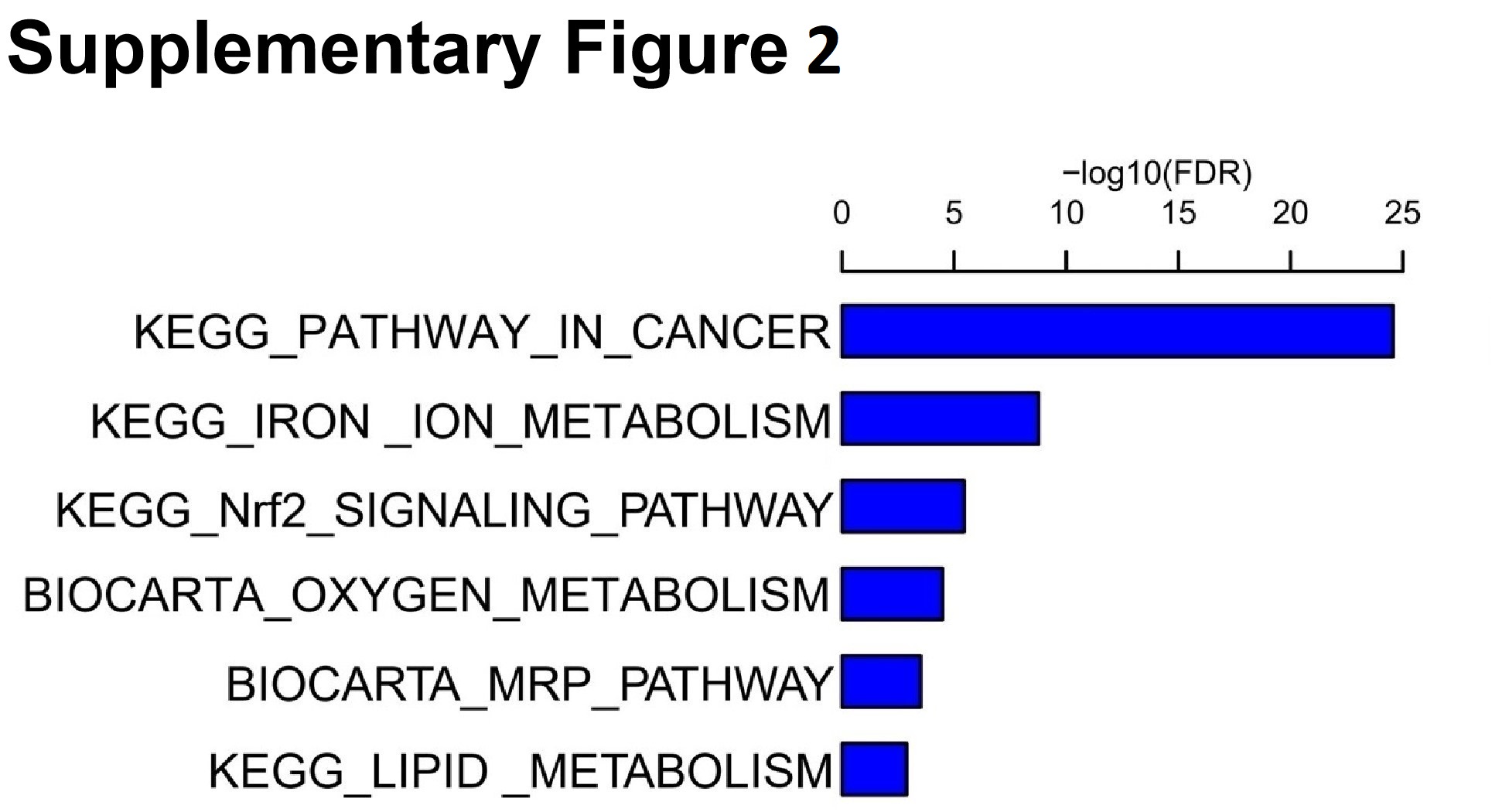

Supplement: Supplementary Figure 2 — KEGG pathway enrichment analysis for these DEGs. [file Image2.jpeg]

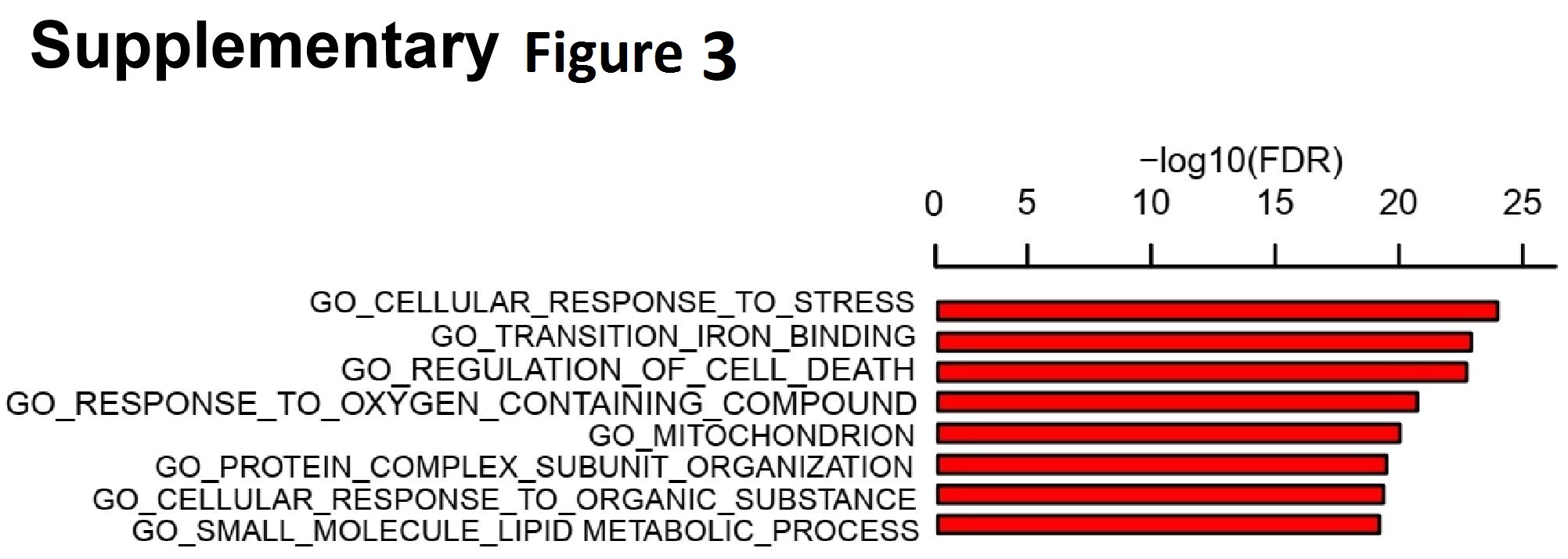

Supplement: Supplementary Figure 3 — GO functional analyses for these DEGs were performed based on the GSEA database. [file Image3.jpeg]

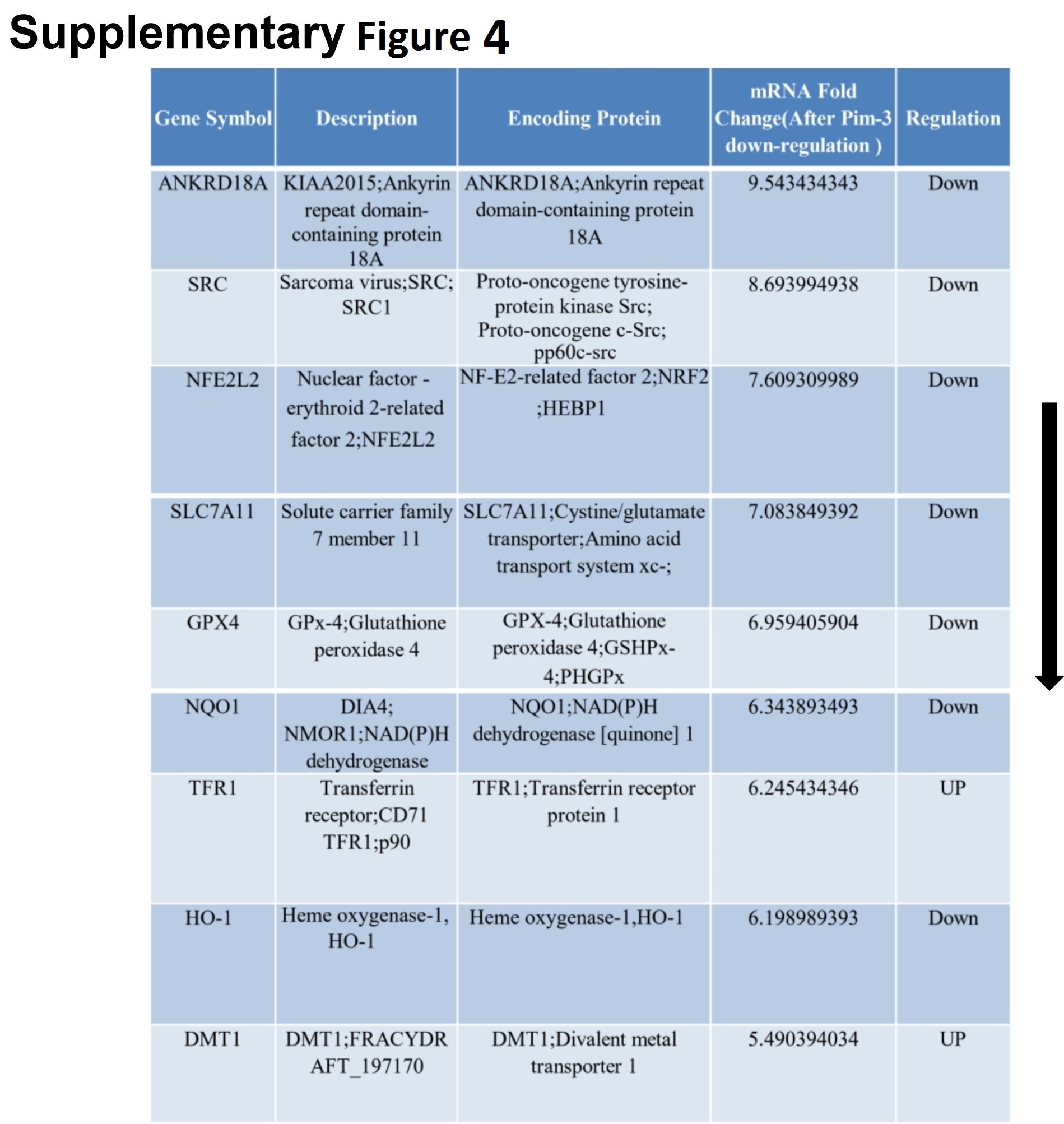

Supplement: Supplementary Figure 4 — The expression fold changes and encoded proteins of the indicated DEGs were presented. [file Image4.jpeg]

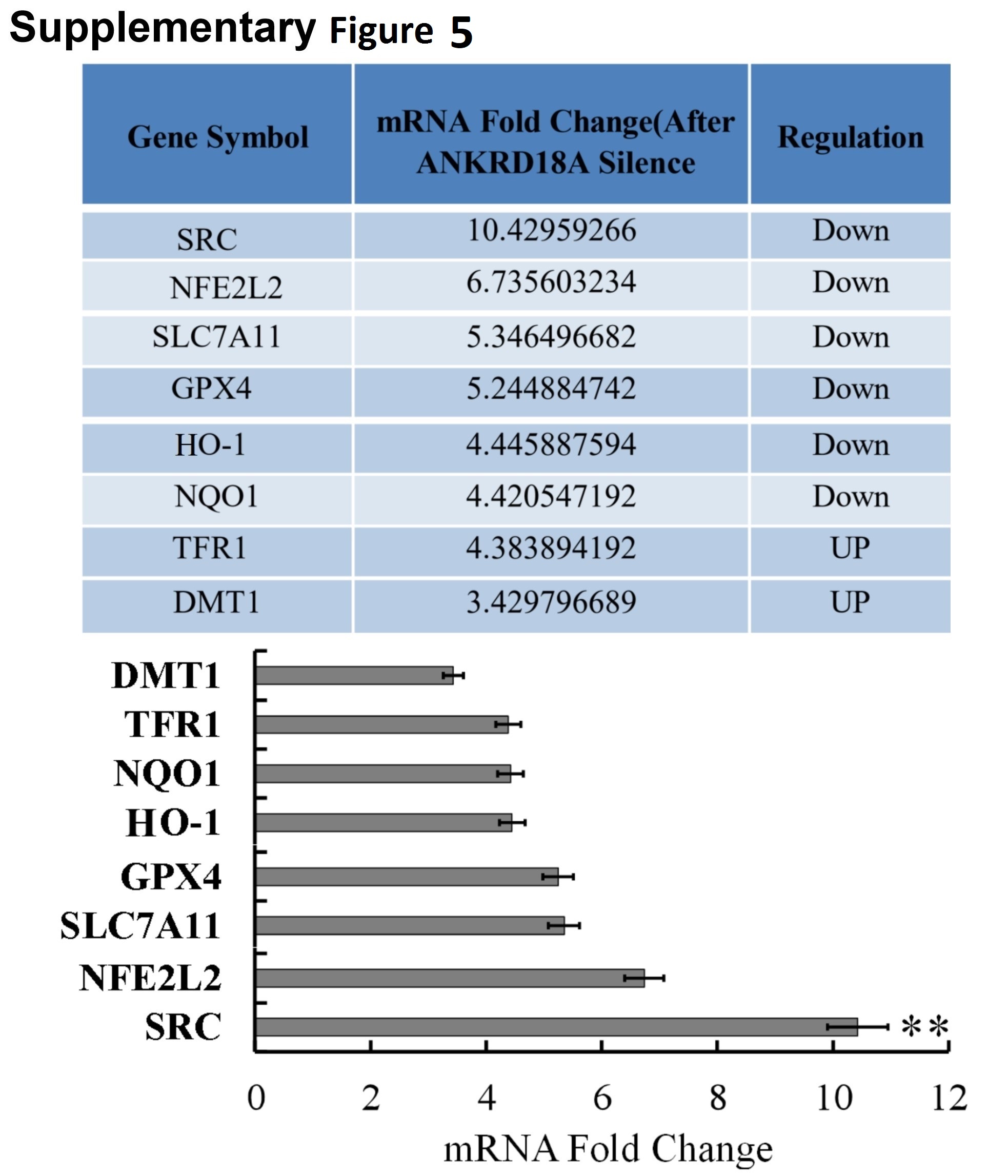

Supplement: Supplementary Figure 5 — Huh7/SOR cells were transfected with plasmids containing shRNAs targeting the ANKRD18A gene. The mRNA expression levels of SRC, NFE2L2, SLC7A11, GPX4, HO-1, NQO1, TFR1 and DMT1 were determined via HCS. The experiments were repeated three times, and the representative results are expressed as the means ± SD. **P<0.01 versus the control. [file Image5.jpeg]

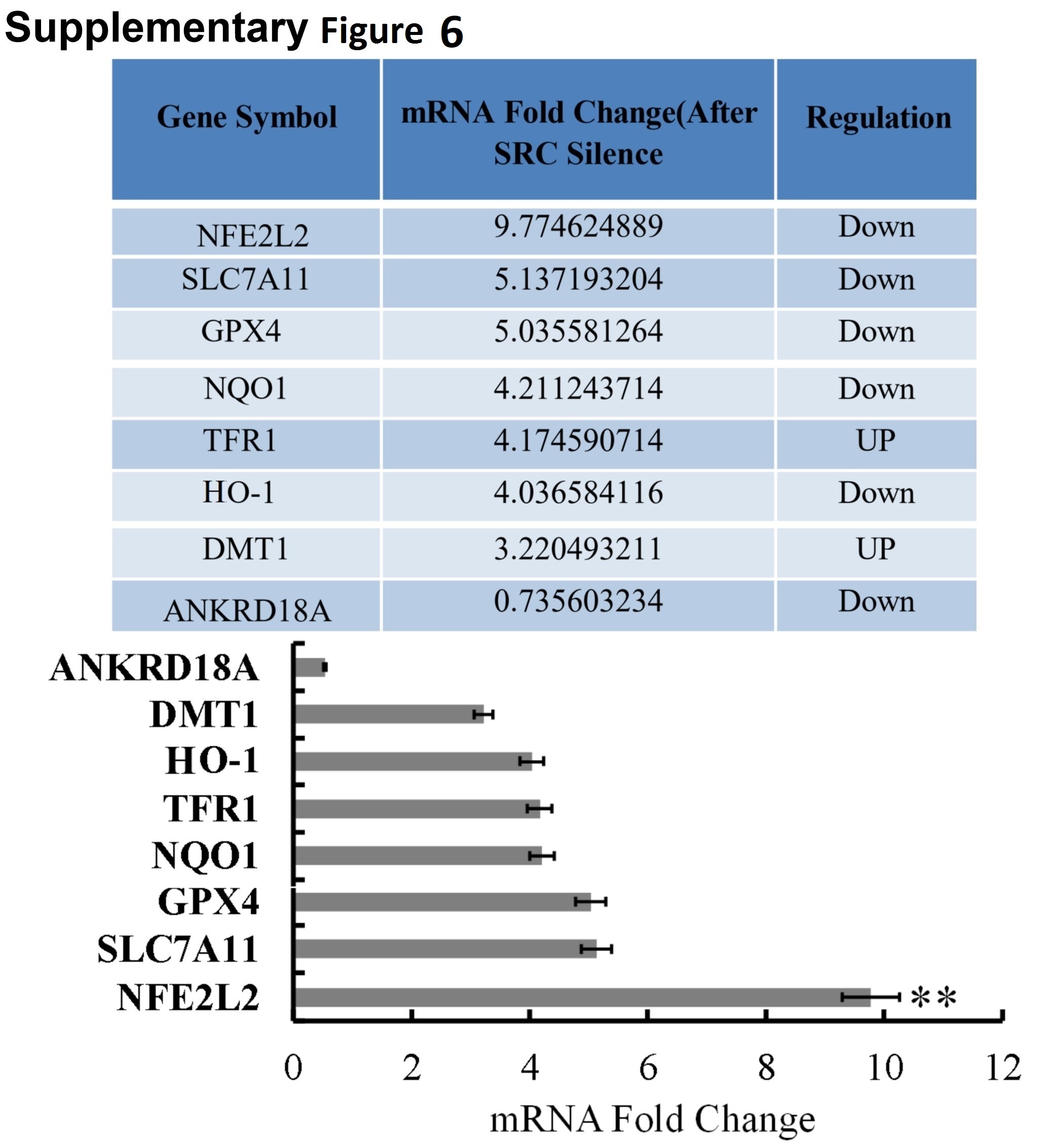

Supplement: Supplementary Figure 6 — Huh7/SOR cells were transfected with plasmids containing shRNAs targeting the SRC gene. The mRNA expression levels of NFE2L2, SLC7A11, GPX4, NQO1, TFR1, HO-1, DMT1 and ANKRD18A were determined via HCS. The experiments were repeated three times, and the representative results are expressed as the means ± SD. **P<0.01 versus the control. [file Image6.jpeg]
